# Death from COVID-19 in contexts of social deprivation in Mexico

**DOI:** 10.3389/fpubh.2024.1463979

**Published:** 2024-10-09

**Authors:** Oscar A. Martínez-Martínez, Araceli Ramírez-López, Brenda Coutiñho, Javier Reyes-Martínez

**Affiliations:** ^1^Department of Social and Political Sciences, Universidad Iberoamericana, Ciudad de México, Mexico; ^2^Colegio de Postgraduados, Montecillo, Mexico; ^3^Universidad Nacional Autónoma de México, Centro Regional de Investigaciones Multidisciplinarias, Cuernavaca, Morelos, Mexico; ^4^División de Administración Pública, Centro de Investigación y Docencia Económicas (CIDE), Ciudad de México, Mexico

**Keywords:** poverty, social deprivations, COVID-19, death, Mexico

## Abstract

**Introduction:**

Poverty is one of the macro factors that has been little studied in terms of its effect on death from COVID-19 since most studies have focused only on investigating whether the pandemic increased poverty or not. With that on mind, the present study aims to analyze how the social deprivations that comprise the measurement of municipal poverty in interaction with health comorbidities and sociodemographic characteristics, increased the probability of death from COVID-19.

**Methods:**

The study is cross-sectional and covers daily reports on the conditions of COVID-19 in the Mexican population for almost 2 years. Using data from the National Epidemiological Surveillance System and the National Council for Evaluation of the Social Development Policy (*N* = 5,387,981), we employ a Generalized Linear Mixed Model (GLMM), specifically a binomial generalized linear mixed model.

**Results:**

The findings indicate that, besides comorbidities, sociodemographic traits, and clinical aspects, living in a municipality where one or more of the social deprivations exist increases the probability of death. Specifically, in those municipalities where there is deprivation in education, social security, and food, as well as deprivation due to access to health services and deprivation in household services, the probability of death was greater.

**Discussion:**

Living in a municipality with one or more of the social deprivations that compose poverty generated a greater probability of death. Each one of them or together, shows that poverty is a substantial factor for a pandemic like COVID-19 to worsen contagion and death, becoming a circle from which it is difficult to escape.

## 1 Introduction

The first case of COVID-19 was recorded in December 2019 in Wuhan China, when a group of people with pneumonia of unknown origin was reported; some days later, the Chinese health authorities confirmed that it was COVID-19 or SARS-CoV2 (1). From that moment on, countries around the world began to prepare for this health emergency, implementing various measures, such as quarantines and social distancing (2) as well as prevention measures such as the use of face masks, application of antibacterial gel, and hand washing frequently, in order to prevent the countries’ health systems from collapsing (3). Despite the various actions of governments, there were 676,609,955 infected people globally and 6,881,955 deaths from this disease (4).

On May 5, 2023, the World Health Organization officially ended the COVID-19 pandemic, however, this does not mean that this virus has ceased to be a threat to global health as this same organization points out. Therefore, it is necessary to investigate which health and social factors mainly influenced people’s deaths. In this regard, different investigations found that certain comorbidities were related to this disease (5) and increased the probability of death from SARS-CoV2 (6), putting some populations at greater risk of developing complications (7). Within these comorbidities are cardiovascular diseases, diabetes, hypertension, obesity, and Chronic Obstructive Pulmonary Disease (COPD) (7, 8).

Health comorbidities had different implications in infected people. It has been found that patients with a greater number of comorbidities had less encouraging diagnoses (9), were more likely to develop severe symptoms (10), and therefore, were more likely to enter hospital intensive care (11). Likewise, other factors such as age and sex significantly changed the probability of death (8), thus older adults and men were within the group with the highest probability of death (3).

Some studies have shown that besides comorbidities and sociodemographic variables, there are social determinants of health that place certain populations at greater risk, which are determined by socioeconomic conditions that reproduce inequalities and vulnerability (12, 13), such as income, education, housing, working conditions, food insecurity, and discrimination (14, 15), which are present mainly in populations in poverty (16). In that sense, poverty and its relationship with the COVID-19 pandemic is an understudied aspect. In a few studies, evidence show that poverty was a factor that contributed to the spread of the pandemic due to inequality in access to health services and treatments of this disease (17). Similarly, they found that income was a gradient that was linked to excess mortality from COVID-19 in the poorest municipalities (18). This could be explained by the lack of income and the difficulty or impossibility of social isolation due to the dynamics and conditions of the labor market (17) of people in conditions of poverty. For these reasons, people with higher levels of poverty had lower survival rates from the virus compared to less poor people (19).

Other evidence indicates that municipalities with greater poverty had a higher density of COVID-19 cases (20). Hence, populations with better access to drinking water, sanitation, electricity, food, and private insurance, (i.e., these aspects are part of deprivation in multidimensional poverty measurements) had a greater capacity to prevent contagion (21) and death from this virus.

Poverty is one of the macro factors that has been little studied in terms of its effect on death from COVID-19 since most studies have focused on investigating whether the pandemic increased poverty (22–24) or not. Therefore, our findings cover gaps in the literature for countries that have high levels of poverty such as Mexico, where, during the first 2 years of the pandemic, 37.9% of people were in poverty and 6.7% in extreme poverty (25). Regarding the dimensions that make up this multidimensional measurement, the population presented different deprivations: 52% in social security, 28.2% in health services, 19.2% in education, 17.9% in housing services, 9.3% in quality and spaces of the housing, likewise 22.5% of the population were food insecure and 17.2% did not have enough income to buy a basic food basket for adequate nutrition (25). Although in the last poverty measurement, there was a reduction in poverty, 31.1% of the population continues in these conditions (26) in a country of more than 100 million people.

Under this context of social deprivation and poverty, the data on the COVID-19 pandemic show that there were 7,587,643 infections and 333,913 deaths from this virus, until May 5, 2023 (27). Therefore, the question we seek to answer is: how did the social deprivations that comprise the municipal poverty measurement affect the probability of death from COVID-19? The research is relevant because the challenge of facing a pandemic such as COVID-19 requires studying in a multidimensional way that includes both health and social vulnerabilities. Above all, different governments, especially those in the global south, are going through a series of historical problems related to poverty, marginalization, and vulnerability (28) as well as the lack of a strong public health systems, which allows us to face the epidemiological challenges of the population (29).

Bearing that on mind, the present study aims to analyze how the social deprivations that comprise the measurement of municipal poverty, in interaction with health comorbidities and sociodemographic characteristics, increased the probability of death from COVID-19.

## 2 Methods

### 2.1 Database

The information was obtained from two open-access databases, one prepared by the National Epidemiological Surveillance System published by the General Direction of Epidemiology belonging to the Ministry of Health of Mexico, the second is the 2020 municipal poverty measurement, carried out by the National Council for Evaluation of the Social Development Policy (CONEVAL, in Spanish). Regarding the first database, it is where COVID-19 cases have historically been recorded in the country since April 2020. Each state is responsible for monitoring cases and reporting to the Federal Ministry of Health. This dataset includes data from COVID-19 patients who voluntarily underwent SARS-CoV-2 testing, both in public and private hospitals and clinics, as well as in laboratories. The database records confirmed, negative, and suspected cases, from different municipalities in the country, as well as their age, sex, ethnicity, and comorbidities (27).

At the end of January 2022, 14,814,144 cases were registered in the database, of which 8,819, 422 were negative, 5,416,636 were positive and 641,078 were suspicious. We built our database with people who tested positive for COVID-19. From these, we excluded those observations that had missing values in three or more variables. The database consisted of a final sample of 5,387,981 cases confirmed with COVID-19, on which the information from the second database was imputed, which contained the percentages at the municipal level of each of the dimensions that make up the poverty measurement (30), in such a way that the seven deprivations of the multidimensional measurement of poverty were associated with the municipality of residence of each observation (see Table 1).

**Table 1:** Description of dependent and independent variables.

Variables	Explanation	Values
Dependent variable		
Death from COVID-19	Indicates whether the patient died from COVID-19 or not.	1 = Death
		0 = Otherwise
Independent variables		
Sex	Biological distinction that classifies people into men or women.	1 = Female
		0 = Male
Age	Identifies the patient’s age.	Continuous variable
Indigenous	Identify if the person considers themselves indigenous.	1 = Indigenous
		0 = Otherwise
Hypertension	Identifies whether the patient has a diagnosis of hypertension.	1 = Has hypertension
		0 = Otherwise
Diabetes	Identifies whether the patient has a diagnosis of diabetes.	1 = Has diabetes
		0 = Otherwise
Obesity	Identifies whether the patient has a diagnosis of obesity.	1 = Has obesity
		0 = Otherwise
Chronic kidney disease	Identifies whether the patient was diagnosed with chronic kidney disease.	1 = Chronic kidney disease
		0 = Otherwise
Pneumonia	Identifies whether the patient was diagnosed with pneumonia.	1 = If pneumonia is present
		0 = Otherwise
HCMP	Identifies the type of care the patient received on the medical unit. It is labeled as outpatient if he/she returned home or is labeled as hospitalized if he/she was admitted to the hospital.	1 = If the case is ambulatory
		0 = If the patient is hospitalized
Intubated	Identifies if the patient required intubation or not.	1 = Intubated patient
		0 = Otherwise
Deprivation in education	Percentage of inhabitants in the municipality who have a lack of education.	Continuous variable
Deprivation in access to social security	Percentage of inhabitants in the municipality who have lack of access to social security.	Continuous variable
Deprivation in access to health services	Percentage of inhabitants in the municipality who have a lack of access to health.	Continuous variable
Deprivation in quality spaces in the household	Percentage of inhabitants in the municipality who have a lack in the quality of housing spaces.	Continuous variable
Deprivation in household services	Percentage of inhabitants in the municipality who have a lack of home services.	Continuous variable
Deprivation in access to food	Percentage of inhabitants in the municipality who have food insecurity.	Continuous variable
Deprivation in income	Percentage of inhabitants in the municipality who live in households with income below the poverty line by income.	Continuous variable

Source. Author’s elaboration.

### 2.2 Measure

The variables used in the study are found in Table 1. Death from COVID-19 is determined by the Ministry of Health as part of the death monitoring subject to epidemiological surveillance (31). The independent variables are divided into four blocks, the first included the comorbidities evidenced in different studies that have shown a strong association with the probability of death from COVID-19, such as diabetes, hypertension, obesity, pneumonia, and kidney diseases (6, 32). The second is the risk conditions, such as age, sex, and being indigenous, which are based on different previous research that points to their association with death from the virus (11, 33, 34).

The third comprised clinical variables since as documented (35) the treatment of manifestations due to the SARS-CoV-2 coronavirus is in relation to the severity of the symptoms and the possibility of access to hospitals equipped to treat the disease. In this case, the variables used were Health Care Management of Patients (HCMP) and whether the person was intubated or not. The fourth block included the seven dimensions that make up the multidimensional measurement of poverty: deprivation in social security, health services, education, housing services, quality and spaces of housing, income, and food insecurity (36).

### 2.3 Procedures and data analysis

The employed model is the Generalized Linear Mixed Model (GLMM), specifically a binomial generalized linear mixed model, expressed by the equation


$$\begin{array}{l} \ln\left[\frac{\pi_{ij}}{1-\pi_{ij}}\right]=\beta_{0}+\overset{10}{\underset{k=1}{\sum }}\beta_{k}{x_{ij}}^{\left(k\right)}+\overset{17}{\underset{k=11}{\sum }}\beta_{k}z_{i}^{\left(k\right)}\\ +u_{i}\;\mathrm{para}\;i=1,2,\ldots ,M\;y\;j=1,2,\ldots ,n_{i} \end{array}$$

On the left side of the equation, the link function is equal to the logit function $\mathrm{logit}\left(\pi_{ij}\right)$ , where $\pi_{ij}$ represents the probability of death for individual j in municipality i. It’s worth noting that $\frac{\pi_{ij}}{1-\pi_{ij}}$is known as the odds of the probability of death. On the right side of the equation, there is the linear predictor, consisting of both fixed and random effects. The first part is a linear combination of regression coefficients ${\left\{\beta_{k}\right\}}_{k=0}^{17}$ with ${\left\{{x_{ij}}^{\left(k\right)}\right\}}_{k=1}^{10}$and ${\left\{z_{i}^{\left(g\right)}\right\}}_{g=11}^{17}$, where ${x_{ij}}^{\left(k\right)}$ representing the covariate $\left(k\right)$ associated with individual j in municipality i and $z_{i}^{\left(g\right)}$being the covariate $\left(g\right)$ associated with municipality i. In other words, the fixed part captures the effect of covariates at the individual level and some variables at the municipal level. The random effects part of the model refers to the term $u_{i}$, and its existence assumes that specific conditions in each municipality, not accounted for in the variables $z_{i}^{\left(g\right)}$, may influence the variability of the probability of death. M and $n_{i}$ represent the number of municipalities and the number of infected individuals per municipality, respectively.

The selection of the model employed the Bayesian Information Criterion ($BIC=Rln\left(N\right)-2\ln\left(L\right)$) and the Akaike Information Criterion (AIC) $AIC=2R-2\ln\left(L\right)$, where R is the number of parameters estimated, L is the likelihood model, and N is the number of observations. In order to evaluate the goodness of fit, the log-transformed residual goodness of fit statistic (37) was used and is defined by $T=\frac{S-E\left[S\right]}{Var\left[S\right]}$, where $S={\hat{\varepsilon }}^{´}\hat{\varepsilon }$, $\hat{\varepsilon }=-\ln\left(1-|e|\right)$, e is the estimated residual vector and under the null hypothesis, T has standard normal distribution. In this model $T=1.32<z_{0.95}$, with critical value $z_{0.95}=1.64$.

## 3 Results

Table 2 shows the main characteristics of the people in the study, specifically the percentages of deaths from COVID-19, sociodemographic data, hospitalizations, intubated people, health comorbidities, and the seven social deprivations that compose poverty at the municipal level.

**Table 2:** Characteristics of the population.

Variables	*Yes %*	*Not %*
Death from COVID-19	5.6	94.4
Hypertension	12.8	87.2
Diabetes	9.6	90.4
Obesity	10.7	89.3
Chronic kidney disease	1.1	98.9
Pneumonia	8.7	91.3
Indigenous	0.94	99.06
HCMP	11.9	88.1
Intubated	1.4	98.6
Deprivation in education	12.2	87.8
Deprivation in access to social security	49.2	50.8
Deprivation in access to health services	26.6	73.4
Deprivation in quality spaces in the household	6.65	93.35
Deprivation in household services	9.29	90.71
Deprivation in access to food	18.6	81.4
Deprivation in income	44.9	55.1
Sex Male: 47.96% Female: 52.04%		
Age (mean): 40.24		

Source. Author’s elaboration, from the MMH (27).

Table 3 shows the results of the GLMM model. Regarding sex, when the person with COVID-19 is a woman, she is 0.7321 times less likely to die than when she is a man. In relation to age, the odds of the probability of death are 1.046 times greater for each additional year of age; in that sense, the probability of death is 1.252 times greater for each additional 5 years of age. Being indigenous increases the probability of death by 1.063 times compared to those who are not.

**Table 3:** Probabilities of death from COVID-19.

Model fixed effects:					
Variable	Coefficient $\beta_{k}$ (Std Error)	*Z*	*p* value	Odds ratio $e^{\beta_{k}}$	95% Conf. Interval
Sex	−0.312 (0.012)	−25.783	<0.001	0.732****	(−0.336,-0.288)
Age	0.045 (0.000)	111.315	<0.001	1.046****	(0.044, 0.046)
Indigenous	0.062 (0.040)	1.549	0.063	1.063*	(0.000, 0.930)
Hypertension	0.133 (0.014)	9.638	<0.001	1.142****	(0.106,0.159)
Diabetes	0.182 (0.014)	13.267	<0.001	1.199****	(0.155,0.209)
Obesity	0.214 (0.015)	13.975	<0.001	1.238****	(0.184,0.244)
Chronic Kidney Disease	0.617 (0.027)	22.828	<0.001	1.853****	(0.564,0.670)
Pneumonia	0.807 (0.014)	59.242	<0.001	2.241****	(0.781,0.834)
HCMP	4.228 (0.023)	186.861	<0.001	68.579****	(4.184,4.273)
Intubated	2.095 (0.024)	87.133	<0.001	8.125****	(2.048,2.142)
Deprivation in quality spaces in the household	0.007 (0.002)	2.884	0.004	1.007***	(0.003,0.013)
Deprivation in household services	0.002 (0.001)	1.54	0.063	1.002*	(0.000,0.005)
Deprivation in income	0.006 (0.001)	5.554	<0.001	1.006****	(0.003,0.008)
Deprivation in access to food	0.006 (0.004)	1.42	0.077	1.006*	(0.002, 0.013)
Deprivation in access to social security	0.008 (0.001)	6.234	<0.001	1.008****	(0.006,0.011)
Deprivation in access to health services	0.006 (0.004)	1.71	0.043	1.006**	(0.001, 0.012)
Deprivation in education	0.006 (0.002)	2.625	0.008	1.006***	(0.002,0.011)
Intercept	−7.732 (0.067)	−114.260	<0.001	0.000****	(−7.865, −7.599)
Random effects model:					
Cluster	Coefficient	Variance	Standard deviation	Conf. Interval	
Municipality	Intercept	0.0724	0.269	(0.170,0.385)	
Goodness of fit measures:					
^+^AIC = 704,016, ^++^BIC = 704,709					

Source. Author’s elaboration, from the MMH (27).

^+^Akaike Information Criterion. ^++^Bayesian Information Criterion. ****0.001 significance level. ***0.01 significance level. ** 0.05 significance level. * 0.1 significance level.

In relation to people with health comorbidities, it is found that the probability of death is 1.142 times higher for people who have hypertension compared to those who do not have it. The probability of death with diabetes is 1.199 times greater than that of a person without this disease. Likewise, the probability of death of patients who have obesity is 1.238 times greater than for those without the condition. As for people with chronic kidney disease, the odds of death are 1.853 times greater than people who do not have it. The probability of death of patients who have pneumonia is 2.241 times greater than the probability of those who do not have it. The clinical variables show that the probability of death of people who were hospitalized is 68.579 times greater than the probability of death compared to those who did not require specialized care in a hospital. The odds of death probability increased by 8.125 times when the person was intubated.

Regarding the social deprivations that integrate poverty at the municipal level, their effect is approximately the same magnitude. Results show that living in a municipality where one or more of the social deprivations exist increases the probability of death. Specifically, deprivation in education, income, health services and food, the probability of death for each one is 1.067 times greater for every 10 percentage points when there is any of that deficiency in the municipality. The probability of death related to access to social security is 1.08 times greater for every 10 percentage points when there is this lack in the municipality. The probability of death in patients is 1.07 times greater for every 10 percentage points when there is a deprivation in quality spaces in the household. Having deprivation in household services in the municipality increases the probability of death by 1.02 times for every 10 percentage points.

## 4 Discussion

Different pre-pandemic studies (38, 39) have documented that respiratory viruses are an emerging threat to global health security because they have given rise to epidemics around the world with high morbidity and considerable mortality. COVID-19 is an example of this because it had different negative effects on certain population groups related to sociodemographic conditions, pre-existing comorbidities, as well as the poverty conditions of the context.

Regarding the sociodemographic variables, our study shows that men had a higher probability of death from COVID-19, as other studies have found (40, 41). In the case of age, like other investigations (31, 42), our evidence indicates that as it increases, the probability of death also increases, which is worse in the case of older adults. Self-identify as Indigenous turned out to be a trait that generates a greater probability of death. This is probably due to all the social inequalities that this group historically has experienced (43, 44). For instance, in several studies, it has been observed the lack of access of health services of Indigenous individuals, and how it affects health outcomes (45) and perceptions of health (46).

In the case of health comorbidities, the evidence from our study shows that hypertension, diabetes, obesity, chronic kidney disease, and pneumonia generated a greater probability of death from COVID-19, in the same line as other international studies since the beginning of the pandemic (7, 9, 41, 42). Although having comorbidities was a predominant factor in developing more severe symptoms of the disease (10, 47) and mainly death, our evidence also suggests that being hospitalized for COVID-19 was one of those factors with the highest probability of death. It could be explained by the time (days or weeks) it took to go to the hospital after they had the first symptoms or tested positive, as well as by the lack of access to hospitals in their context or their saturation.

Going to the hospital in an advanced stage of COVID-19 has an aftermath death or more severe diagnoses due to the severity of the disease (35), leading on several occasions to people being intubated, which, according to our evidence, is one of those with the highest probability of death. Some scholars have found (35) that going to the hospital or medical consultation at the beginning of COVID-19 symptoms helped sick people not be hospitalized and be treated on an outpatient basis.

Living in a municipality where one or more of the social deprivations that compose poverty occur generates a greater probability of death. Each one of them or together, shows that poverty is a substantial factor for a pandemic like COVID-19 to worsen contagion and death, becoming a circle from which it is difficult to escape. For example, the probability of dying due to the deprivation of quality spaces in the household can be explained because one of the variables with which this deprivation is integrated is overcrowding; being in a home where several people live in a small space could generate greater contagion among its members. In addition to this, living in a context of deprivation in household services increased the probability of having as an outcome death because it implies not having water and/or drainage inside the home, which made several of the basic recommendations during the pandemic difficult, such as washing hands frequently or washing and disinfecting food and cloths. For this reason, in another investigation, housing conditions worsened the risk of death from coronavirus by up to 63% (48).

During the pandemic, lack of income was one of the main problems due to the loss of both formal and informal employment (49). In addition, some households experienced a temporary reduction in their income, for this reason having this deprivation increased the probability of death, because the lack of economic resources meant that people had to go out and look for work to bring income to the home. In this sense, some studies show that income was directly related to the high number of infections and deaths from COVID-19 (50) and that people in the lowest income decile had a higher probability of dying. by COVID-19 when they were infected compared to those with higher incomes (51).

Regarding food insecurity, both the scarcity of food in the context and the lack of income to buy it, caused people to have to leave their homes in search of food, increasing their probability of contagion and death. Similar studies show that having been infected with COVID-19 and having food insecurity increased the risk of death from this disease (52), which was higher in populations of less than 5,000 inhabitants (40).

In the Mexican case, by not having a formal job, you do not have access to social security and, therefore, to the benefits that it entailed in the time of the pandemic, such as guaranteed salary, teleworking, and health insurance. In this way, the deprivation of social security increased the probability of death. Likewise, having deprivation in health services means that in the context where one lives one does not have access to public or private hospitals or health clinics. Besides, even if health care facilities existed it did not mean that they were prepared to care for COVID-19 patients, given the deficiencies in quality and services in the health system that historically exist in the country (29, 53). For this reason, the lack of access to health services has been considered one of the most important factors impacts on the probability of death from COVID-19 (54), especially due to inequality in access to health services and treatments for this disease (17).

Deprivation in education had effects on death, probably due to the lack of access to information about the pandemic that could have been obtained at the highest levels of education or because of lack of knowledge of their pre-existing health problems and how to treat them, hence other studies have found that educational lag was a predictor of higher mortality from COVID-19 (55, 56).

Our findings show that the social deprivations that make up the measurement of poverty in interaction with pre-existing health comorbidities generated effects on the probability of death, showing that the COVID-19 pandemic was a syndemic—i.e., the combination of two or more simultaneous or successive epidemics in a population, with biological interactions, which worsen the prognosis, as other research has pointed out (51, 57).

## 5 Conclusion

The COVID-19 pandemic showed the need to change the health model focused on corrective care that prevails in the National Health System in Mexico and move to a preventive health policy. In that sense, one of the most important challenges to be considered are modifications in the demographic and epidemiological profile of the country, such as population aging and the increment in comorbidities that rise the probability of death.

Living in contexts of poverty increases the probability of death. Evidence here is especially relevant for countries like Mexico where the majority of its population lives with these conditions. Our finding also has implications in public policy because it shows the relevance of carrying out public policy interventions to reduce the social deprivations that detonate poverty. First, the urgent need to address social inequalities in Mexico, mostly those related to the access of healthcare systems and sanitary conditions. The COVID-19 pandemic underscores the priority of more universal healthcare access to all individuals, regardless of individual conditions or regional contexts.

Second, the central need to move from the health model system focused on corrective care that prevails in the National Health System in Mexico to a preventive health policy. The role of previous comorbidities on the outcomes of the COVID-19 pandemic highlights the importance of healthcare systems that do not ignore the proactive measures for managing chronic conditions, ensuring regular health check-ups, and promoting lifestyle changes to mitigate risks.

## Data Availability

Publicly available datasets were analyzed in this study. This data can be found here: https://www.gob.mx/salud/documentos/datos-abiertos-152127.
